# EEG Oscillations as Neuroplastic Markers of Neural Compensation in Spinal Cord Injury Rehabilitation: The Role of Slow-Frequency Bands

**DOI:** 10.3390/brainsci14121229

**Published:** 2024-12-07

**Authors:** Guilherme J. M. Lacerda, Lucas Camargo, Marta Imamura, Lucas M. Marques, Linamara Battistella, Felipe Fregni

**Affiliations:** 1 Instituto de Medicina Física e Reabilitação, Hospital das Clínicas HCFMUSP, Faculdade de Medicina, Universidade de São Paulo, São Paulo 05403-010, SP, Brazil; guilhermelacerda7@gmail.com (G.J.M.L.); marta.imamura@fm.usp.br (M.I.); linamara@usp.br (L.B.); 2 Neuromodulation Center and Center for Clinical Research Learning, Spaulding Rehabilitation Hospital and Massachusetts General Hospital, Harvard Medical School, Boston, MA 02115, USA; lcamargo@mgh.harvard.edu; 3 Mental Health Department, Santa Casa de São Paulo School of Medical Sciences, São Paulo 01224-001, SP, Brazil; lucasmurrins@gmail.com; 4 Departamento de Medicina Legal, Bioética, Medicina do Trabalho e Medicina Física e Reabilitação do da Faculdade de Medicina da Universidade de São Paulo (FMUSP), São Paulo 01246-903, SP, Brazil

**Keywords:** spinal cord injury, electroencephalogram, biomarkers, neuroplasticity

## Abstract

Background: Spinal cord injury (SCI) affects approximately 250,000 to 500,000 individuals annually. Current therapeutic interventions predominantly focus on mitigating the impact of physical and neurological impairments, with limited functional recovery observed in many patients. Electroencephalogram (EEG) oscillations have been investigated in this context of rehabilitation to identify effective markers for optimizing rehabilitation treatments. Methods: We performed an exploratory cross-sectional study assessing the baseline EEG resting state of 86 participants with SCI as part of the Deficit of Inhibitory as a Marker of Neuroplasticity in Rehabilitation Cohort Study (DEFINE). Results: Our multivariate models demonstrated a positive correlation between frontal delta asymmetry and depression symptoms, while the frontal alpha asymmetry band and anxiety symptoms were negatively correlated. Theta oscillations were negatively associated with motor-evoked potential (MEP), whereas alpha oscillations were positively associated with MEP in all regions of interest and with CPM response as a negative correlation. Based on the potential role of lower-frequency oscillations in exerting a salutogenic compensatory effect, detrimental clinical and neurophysiological markers, such as depression and lower ME, likely induce slow oscillatory rhythms. Alpha oscillations may indicate a more salutogenic state, often associated with various cognitive functions, such as attention and memory processing. Conclusions: These results show an attempt by the CNS to reorganize and restore function despite the disruption caused by SCI. Indeed, this finding also challenges the notion that low-frequency EEG rhythms are associated with cortical lesions. These results may contribute to the development of rehabilitation strategies and potentially improve the clinical outcomes of patients with SCI.

## 1. Introduction

Spinal cord injury (SCI) affects approximately 250,000 to 500,000 individuals every year, with violence and motor vehicular crashes (MVCs) as the main causes of this condition; however, nontraumatic etiologies are still relevant, such as neurodegenerative and malignant conditions [1]. Recovering and assessing SCI patients is challenging, owing to the heterogeneity of etiology, severity (level lesion, partial or complete), and population affected [2,3]. SCI affects young adults (less than 30 years old) the most and has a strong lifetime economic impact of between $2 and 4 billion [4]. Current treatments focus on reducing the impact of physical and neurological disabilities with limited functional recovery in most patients [5]. This condition not only causes physical disability but also negatively affects patients’ self-esteem, increasing the risk of psychiatric conditions, mortality rate, and the costs of health care for family members and the public system [6,7]. For this reason, understanding the neurophysiological mechanisms and neuroplasticity changes after SCI could help the development of more accurate neurological tests and improve the treatment options in the physical medicine and rehabilitation field.

After an injury to the central or peripheral nervous system, the brain is naturally designed to adapt by strengthening or weakening connections between certain groups of neurons. This adaptive process helps preserve functionality and maintain homeostasis within the nervous system [8]. However, traumatic and nontraumatic injuries can affect these compensatory mechanisms by disrupting the motor and somatosensory systems [9]. SCI triggers inflammatory responses, including apoptosis and necrosis, acute axonal degeneration, axonal remodeling, demyelination, and glial scar formation [6]. Owing to the complexity of this condition, previous researchers have investigated the use of neurophysiological tests, such as electroencephalogram (EEG), transcranial magnetic stimulation (TMS), and conditioned pain modulation (CPM), to identify potential biomarkers for neuroplasticity and imbalanced cerebral activities in patients with functional disabilities [10].

EEG is extensively used in medicine to diagnose epilepsy and sleep disturbances, and its applications have been explored to help patients with many other neuropsychiatric conditions and disabilities [11]. Vuckovic et al. [12] observed significant differences in alpha power between different groups of patients with spinal cord injury and neuropathic pain, with patients who developed pain presenting a reduced alpha power resting state in the parietal region compared to those who did not develop pain. Moreover, Wang et al. [13] demonstrated lower alpha power in SCI patients with neuropathic pain than in a group with only numbness, but this difference was identified across multiple brain regions, such as the frontal, motor, and parietal regions. Furthermore, Wang et al. [13] demonstrated that SCI could lead to functional sensory and metabolic changes in the frontal, premotor, and thalamic regions.

Some authors hypothesize that SCI induces a shift in spectral oscillations towards low-frequency bands (such as theta oscillations) caused by a thalamocortical dysrhythmia (TDC) [14,15,16]. Additional findings included the observation of reduced alpha and increased beta power in SCI patients [17,18]. In our studies involving patients with SCI, we observed a reduction in alpha and theta activity in patients with neuropathic pain and SCI compared to healthy controls during the EEG resting state, and the alpha/theta ratio is a potential surrogate of functional recovery during rehabilitation [19,20]. In our EEG studies in stroke, knee osteoarthritis, and fibromyalgia patients, we observed higher theta activity as a potential compensatory mechanism of pain and functional recovery, which brings attention to TDC in those populations as well [21,22,23,24]. In addition, frontal asymmetry in alpha and low-frequency bands, such as delta and theta bands, has been studied as a metric of emotion, motivation, and psychiatric conditions in both resting-state and task conditions [25,26].

However, other investigations showed an increase in EEG resting-state theta and alpha power in SCI patients [27,28]. The role of EEG oscillations in SCI recovery and the mechanism of neuroplasticity after the lesion is not completely clear. Given these mixed results regarding neural oscillations and their relationship with SCI, our cohort combined EEG measures with assessments of clinical improvement to investigate potential biomarkers of neuroplasticity. Additionally, while CPM assesses pain processing and the descending pain inhibitory system, TMS provides critical parameters of cortical excitability, including motor threshold (MT), motor-evoked potential (MEP), intracortical inhibition (ICI), and intracortical facilitation (ICF), thereby enhancing neurophysiological test interpretation when combined with EEG signals [21].

Based on the evidence in the literature and the nature of this exploratory cross-sectional study, we hypothesized that EEG oscillations such as delta, theta, and alpha bands are associated with clinical and other neurophysiological variables in patients with SCI. Low-frequency EEG oscillations are potential biomarkers of motor recovery and psychological status, indicating a potential salutogenic compensatory mechanism in the central nervous system. These associations involve factors such as cognitive–emotional status, neurological impairment, lesion duration and level, conditioning pain tests, and cortical excitability parameters. Unfortunately, there is a lack of neurophysiological tests available to assess motor recovery in SCI in clinical practice, but EEG has potential applications that need to be explored in the physical rehabilitation field.

## 2. Materials and Methods

### 2.1. Study Design and Participants

We performed a cross-sectional study assessing only the baseline data of 102 participants with SCI admitted to the “Instituto de Medicina Física e Reabilitação” (IMREA), Brazil, as part of the Deficit of Inhibitory as a Marker of Neuroplasticity (DEFINE study) in Rehabilitation: A Longitudinal Cohort Study Protocol project [29]. This project is approved by Hospital das Clínicas da Faculdade de Medicina da Universidade de São Paulo Ethics Committee. All participants signed an informed consent form according to the Declaration of Helsinki [30].

The inclusion criteria were as follows: (i) subjects between 18 and 65 years old, (ii) clinical and radiological diagnosis of traumatic SCI, (iii) a duration between 1 and 36 months after the lesion, (iv) American Spinal Injury Association Impairment Scale (AIS) between the “A” and “D” classification, and (v) statable clinical and cognitive status. The exclusion criteria were as follows: (i) previous history or concomitant neurological conditions related to SCI, (ii) history of orthopedic conditions or unhealed lower limb fractures, and (iii) presence of tracheostomy.

### 2.2. Demographic, Clinical, and Neurophysiological Variables

We collected demographic information from participants, such as age, biological sex, body mass index (BMI), clinical variables such as level and time of lesion, validated scales such as the emotional (Hospital Anxiety and Depression Scale) Montreal Cognitive Assessment (MOCA) and AIS, and other neurophysiological tests such as transcranial magnetic stimulation (TMS) and conditioned pain modulation (CPM). The assessment methodology and data collection can be found in the study protocol [10].

### 2.3. EEG Resting-State Preprocessing

From the 102 participants in this study, we collected EEG baseline data from 86 subjects using an ANT Neuro 64-channel EEG system (ANT Neuro, Enschede, The Netherlands) describing results from the resting-state period: 5 min with eyes opened and 5 min with eyes closed. However, we considered only the period with eyes closed in this analysis to minimize external visual stimuli and eye movements.

We followed the pre-processing method described in our cohort study protocol [10]. We preprocessed the original data using EEGLab in MATLAB (MATLAB R2023a, MathWorks Inc., Natick, MA, USA, 2023) to remove any potential artifacts. We followed the preprocessing pipeline proposed by Makoto [31] using the Darbeliai EEGLAB plugin following these steps: (i) bandpass of 1 Hz (High Pass) and 50 Hz (Low Pass), (ii) downsampling from 1000 Hz to 250 Hz, (iii) re-referencing the channels using the electrode average, and (iv) 60 Hz power line noise correction (frequency in the United States).

The data were visually inspected, and channels containing artifacts were rejected before performing the independent component analysis (ICA), eliminating channels that (i) were flat for longer than three seconds, (ii) showed high-frequency noise greater than two standard deviations, and (iii) showed correlation with neighboring channels lower than 0.8 using the Clean_rawdata EEGLAB plugin (v2.2). The remaining channels were fed into the Infomax ICA calculation using the Darbeliai plugin to identify artifacts effectively [32,33]. With the ICLabel toolbox, we could remove components associated with heart rate, muscle noise, blinking, and eye movement [34].

Finally, we used the pop_spectopo EEGLab function with fast Fourier transformation with 2s windows and 50% overlap. The relative power was calculated for the following bands: delta (1–3.9 Hz), theta (4–7.9 Hz), alpha (8–12.9 Hz), and beta (13–30 Hz), as well as for the sub-bands low alpha (8–9.9 Hz), high alpha (10–12.9 Hz), low beta (13–19.9 Hz), and high beta (20–30 Hz), from the following regions of interest (ROIs): the frontal, central, and parietal areas. More details can be found in our cohort study protocol [10].

### 2.4. Statistical Analysis

We performed a descriptive analysis of demographic, clinical, and neurophysiological data using the mean and standard deviation (SD) for continuous variables, as well as the sample size and percentage for binary or categorical variables. We used STATA^®^ 17.0 for all statistical analyses. More details are described in Appendix A.

## 3. Results

### 3.1. Demographic and Clinical Variables

One hundred and two patients with SCI were included in this study, with a mean age of 41 years (SD: 16), eighty males (87.9%) and eleven females (12.1%), forty-one were white (45.1%), ten were black (11%), thirty-nine were of mixed race (42.9%), and one was indigenous (1.0%). Participants who self-reported as “pardo” in Portuguese were included in the “mixed race” category. In addition, we observed that the lesion levels of our participants were forty-eight cervical (47.06%), forty-one thoracic (40.21%), twelve lumbar (11.76), and one sacral (1.0%); forty-five had tetraplegia (44.12%), and fifty-seven paraplegia (55.88%).

Additional demographic and clinical data are shown in Table 1. Continuous variables are presented with their mean and SD, while categorical or binary variables are presented with their *n* and percentage.

### 3.2. Neurophysiological Findings

**Figure 1:**
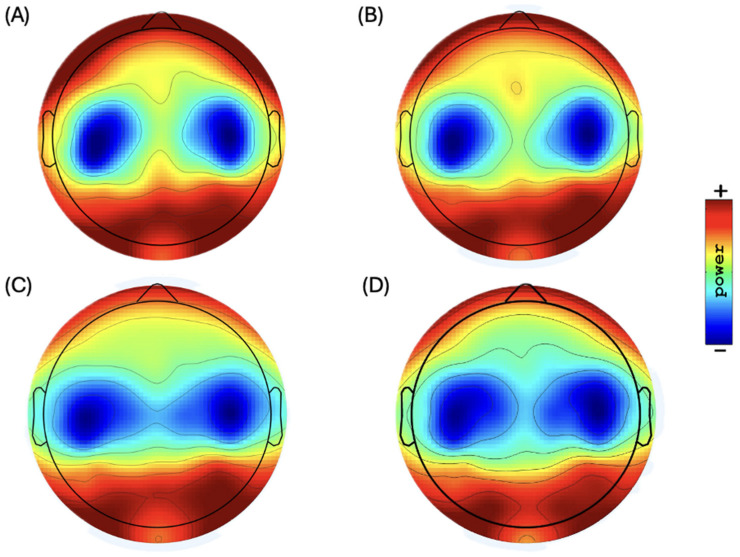
Topographic distribution of scalp plots of EEG bands in resting state: (**A**) delta power, (**B**) theta power (range: 34.5 to 40.0 dB) (10 Ã–log10 P), (**C**) alpha power (range: 35.0 to 42.0 dB) (10 Ã–log10 P), and (**D**) beta power (range: 28.0 to 33.0 dB) (10 Ã–log10 P).

EEG data were recorded from 86 subjects, and the average of their relative power was represented as the mean and SD in each ROI and separated in the right and left brain hemispheres, as displayed in Table 2 and Table 3. Figure 1 shows the topographic distribution of scalp plots in the resting-state EEG.

### 3.3. Univariate Analysis

**Figure 2:**
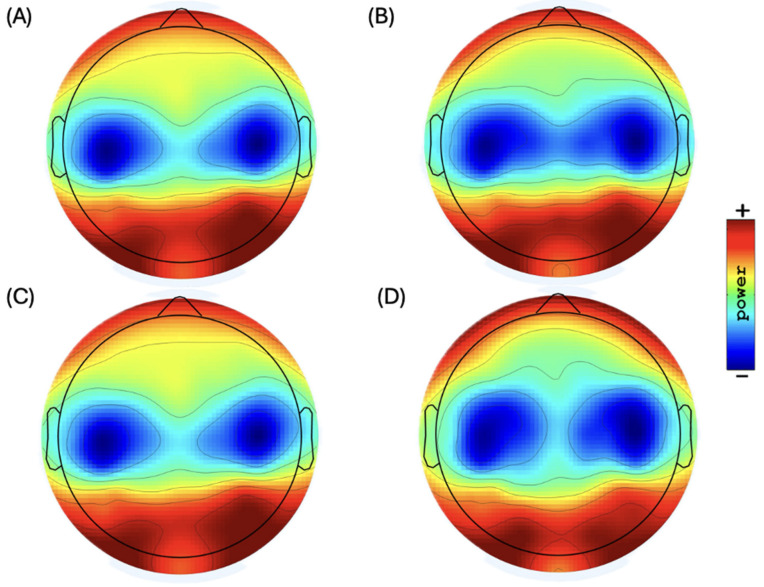
Topographic distribution of scalp plots of EEG sub-bands in resting state: (**A**) low alpha power (range: 37.0 to 45.0 dB) (10 Ã–log10 P), (**B**) high alpha power (range: 35.0 to 41.0 dB) (10 Ã–log10 P), (**C**) low beta power (range: 30.0 to 35.0 dB) (10 Ã–log10 P), and (**D**) high beta power (range: 26.0 to 31.5 dB) (10 Ã–log10 P).

Delta oscillations in the frontal regions demonstrated a negative association with depression symptoms (β-coef.: −0.05, *p*-value: 0.032, 95% CI: −0.01 to 0.00) and a frontal delta asymmetry showed a negative association (towards the right hemisphere) with depression symptoms (β-coef.: −0.11, *p*-value: 0.033, 95% CI: 0.00 to 0.02), demonstrating a reduction of delta frontal activity and a shift towards the right hemisphere as the depression symptoms worsen. A scatter plot of the regression lines is shown in Figure 2.

Theta oscillations demonstrated significant or almost significant negative association with MEP in all ROIs: (i) frontal: β-coef.: −0.04, *p*-value: 0.068, 95% CI: −0.08 to 0.00; (ii) central: β-coef.: −0.03, *p*-value: 0.083, 95% CI: −0.07 to 0.00; (iii) parietal: β-coef.: −0.05, *p*-value: 0.027, 95% CI: −0.09 to −0.01. In contrast, alpha oscillations showed a positive correlation with MEP in all ROIs: (i) frontal: β-coef.: 0.102, *p*-value: 0.002, 95% CI: 0.04 to 0.16; (ii) central: β-coef.: 0.08, *p*-value: 0.008, 95% CI: 0.02 to 0.15; (iii) parietal: β-coef.: 0.106, *p*-value: 0.002, 95% CI: 0.04 to 0.17. These results show an interesting behavior of more activity towards higher frequency bands as the MEP also increases, indicating a potential compensatory mechanism involving theta and alpha oscillations and cortical excitability in patients with SCI.

**Figure 3:**
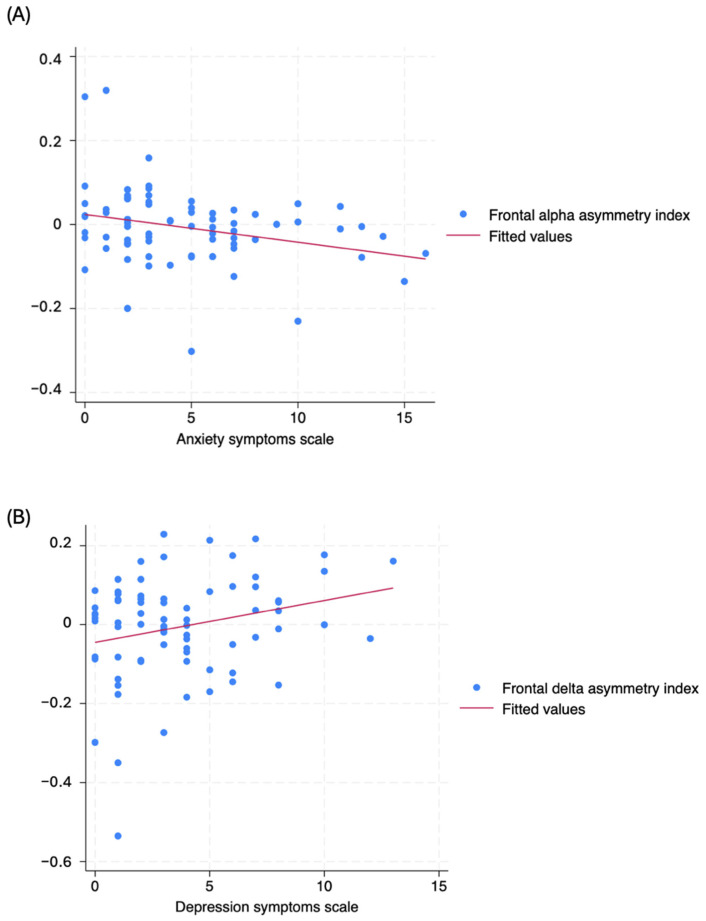
Scatter plot from univariate analysis of (**A**) frontal delta asymmetry index (Y-axis) and depression symptoms scale (X-axis), and (**B**) frontal alpha asymmetry index (Y-axis) and anxiety symptoms (X-axis).

In addition, we observed a negative association between frontal alpha and sleepiness (Î^2^-coef.: −0.01, *p*-value: 0.048, 95% CI: −0.01 to 0.00), as the alpha band is often associated with relaxed wakefulness and inhibitory control in the CNS. Frontal alpha asymmetry demonstrated a negative correlation (towards the left hemisphere) with anxiety symptoms (β-coef.: −0.01, *p*-value: 0.014, 95% CI: −0.01 to −0.00), showing an association between greater alpha activity in the left frontal hemisphere and more severe depression symptoms. Scatter plots are shown in Figure 3.

High alpha oscillations showed a negative relationship with CPM in the right hand in the frontal and parietal regions, with (i) β-coef.: −0.01, *p* = 0.087, 95% CI: −0.02, 0.00 and (ii) β-coef.: −0.01, *p* = 0.044, 95% CI: −0.03, 0.00, respectively, indicating that high alpha oscillations in those regions could be linked to a reduced ability to modulate pain. No significant results were observed for beta oscillations.

### 3.4. Multivariate Analysis

Delta-oscillation models reveal distinct regional effects influenced by various factors. The corresponding model explained the variance with an R-square of 0.25 in the frontal region, with depression showing a negative association with delta-band activity (β-coef.: 0.01, *p*-value: 0.036) and age having a positive and significant relationship (β-coef.: 0.001, *p*-value: 0.035). Other variables, such as the level of lesion, MOCA scores, and years of education, were included to control for their potential impacts on cognition and mental health. In the parietal region, where the model presented an R-square of 0.205, the ICI mean was a significant positive predictor of delta-band activity (β-coefficient: 0.010, *p*-value: 0.022), while age, sex, level of lesion, and lesion time were included to control for their potential impact on neurological impairment. Table 4 presents the delta multivariate models.

Theta-oscillation models showed a significant negative correlation with MEP and an R-square of 0.32, 0.29, and 0.40, respectively, in the frontal (β-coef.: −0.062, *p*-value: 0.034), central (β-coef.: −0.057, *p*-value: 0.049), and parietal (β-coef.: −0.063, *p*-value: 0.021) regions. Age also had a significant negative relationship in the frontal and central areas (β-coef.: −0.105, *p*-value: 0.008). Variables such as biological sex, lesion level, and AIS score were included to control for their potential influence on neurological impairment. Table 5 presents the theta multivariate models.

Alpha-oscillation models showed a significant positive correlation with MEP and R-squares of 0.21, 0.14, and 0.23, respectively, in the frontal (β-coef.: 0.111, *p* = 0.008), central (β-coef.: 0.087, *p* = 0.040), and parietal (β-coef.: 0.110, *p* = 0.010) regions, with age showing a trend toward a negative association in the parietal area (β-coef.: −0.106, *p* = 0.075). Variables such as biological sex, age, lesion level, and lesion time were included to control for their potential influence on neurological impairment. Additionally, the SEPW total scale had a significant negative association with alpha-band activity in the parietal region (β-coefficient: −0.008, *p* = 0.017), and age was a significant negative predictor (β-coefficient: −0.136, *p*-value: 0.012) in this context. Multivariate alpha models are presented in Table 6.

High alpha-oscillation models showed a significant negative correlation with CPM in the right hand, with R-squares of 0.13 and 0.18, respectively, in the frontal (β-coef.: −0.012, *p*-value: 0.049) and parietal (β-coef.: −0.016, *p* = 0.018) regions, and with age also showing a significant negative relationship in the parietal area (β-coef.: −0.148, *p*-value: 0.033). Variables such as sex and lesion level were included to control for their potential influence on brain function. The high alpha multivariate models are presented in Table 7.

Frontal-asymmetry models showed a significant association in the delta band with depression symptoms and lesion time, with an R-square of 0.13, with depression showing a positive relationship with the right hemisphere (β-coef.: 0.010, *p* = 0.036) and lesion time showing a negative relationship (β-coef.: −0.002, *p* = 0.040). The frontal alpha-asymmetry model, with an R-square of 0.13, was negatively correlated with anxiety symptoms (β-coefficient: −0.006, *p* = 0.034) and asymmetry towards the left hemisphere. Variables, such as age, lesion time, and lesion level, were included to control for their potential influence on brain function. Multivariate frontal asymmetry models are presented in Table 8.

A summary of the main results and correlation directions is presented in Table 9. No model was found to be significantly related to beta oscillations.

## 4. Discussion

Our multivariate model results are consistent with those of previous studies, reinforcing the association between EEG oscillations and psychological and other neurophysiological variables. Frontal delta oscillations and asymmetry in the delta and alpha bands are linked to the severity of depression and anxiety symptoms. A reduction in alpha bands and an increase in low-frequency bands in the right hemisphere are often associated with worse psychiatric symptoms. Another interpretation of our results is that the reduction in MEP correlates with higher theta and lower alpha activity across all ROIs. Patients with SCI often present a reduction in their MEP metrics due to impaired motor pathways; therefore, a shift of higher frequency bands to lower frequency bands could result in intrinsic compensatory mechanisms in the cortical–spinal pathway [21]. These results converge with previous findings in the literature that found that the shift towards theta could indicate TDC in this population [14,15,16]. Finally, increased alpha activity in the frontal and parietal regions was associated with a less effective descending inhibitory pain process, as measured in the right hand. However, this relationship was not statistically significant when compared with the left hand.

Anxiety and depression symptoms are prevalent among patients with spinal cord injury (SCI) due to significant functional limitations and a profound impact on quality of life. Beyond these factors, the presence of spinal cord lesions can disrupt compensatory mechanisms in the brain, which may increase the risk of developing mental health issues [9]. The increase in delta activity in the frontal region may be explained by a compensatory mechanism in patients with more depressive symptoms. Low-frequency bands, such as delta oscillations, are associated with homeostatic processes in the cortical and subcortical circuits [35,36]. A recent investigation demonstrated that reduced delta baseline activity is correlated with enhanced efficacy of cognitive behavioral therapy in depression [37]. Moreover, we observed a pattern of frontal delta asymmetry in depression and frontal alpha asymmetry in anxiety (Figure 2). Delta oscillations shift from the left toward the right hemisphere, while alpha oscillations shift from the right toward the left hemisphere as the symptoms worsen, crossing the “x” axis approximately at score five. A closer examination of the behavior of both frequency bands suggests that the reorganization of frontal circuits towards lower frequency bands in the right hemisphere is associated with a greater prevalence of mental health issues.

Other neuroimaging studies have also investigated the use of frontal asymmetry as a biomarker for cognition and psychiatric conditions [38,39,40]. EEG frontal asymmetry scores hold significant potential as tools for understanding cortical activity across various neuropsychiatric conditions in mental health and rehabilitation; however, further research is essential to fully elucidate their role and effectiveness in these contexts. Expanding the evidence will help clarify how these asymmetry scores can be used for diagnosis and treatment in clinical settings, contributing to more targeted and effective interventions.

The reduction of MEP in patients with SCI is expected, as MEP reflects the integrity of descending corticospinal-tract fibers, which are often impaired in patients with SCI [41]. While conditions such as stroke and traumatic brain injury directly affect the cortical substrate, the cortical and subcortical regions remain intact in SCI. Therefore, an injury to the spinal cord triggers cortical reorganization in the brain areas associated with the affected limbs, activating mechanisms of cortical plasticity to promote functional recovery [42]. Cortical reorganization can be attributed to intrinsic connections and inhibitory GABAergic activation, resulting in the plasticity of cortical networks and ICI [43]. The observed negative association between theta oscillations and MEPs and the positive correlation between alpha oscillations and MEP support the theory presented in the literature [21,42,43,44]. This indicates a compensatory mechanism within the CNS, such as changes in theta and alpha oscillations, in an attempt to reorganize and restore function despite the disruption caused by SCI.

Theta oscillations appear to play a compensatory role in response to motor injuries, as demonstrated in previous studies [23,45]. In contrast, alpha oscillations may reflect a more relaxed and salutogenic state, particularly in relation to cognitive functions, such as attention and memory. These distinct patterns of neural activity suggest that different oscillations may indicate various aspects of recovery and brain function post-injury [46]. These associations with MEP are linked to the modulation of the cortical–spinal pathway, while the negative correlation between delta and ICI is explained by an increase in cortical inhibition in the parietal regions, which are involved in cognitive tasks [47,48].

Interestingly, our findings indicate that high alpha oscillations are linked to a lower CPM response, with statistical significance only on the right-hand side. This relationship can be explained by the role of alpha oscillations in modulating cortical excitability and sensory processing, which are essential for the brain’s ability to regulate and inhibit pain; as shown in our CPM metric, a reduced CPM means a worse response to pain [10,49,50]. Additionally, the negative association between the alpha band in the parietal region and sleepiness illustrates the role of alpha activity in cognitive function, fatigue, and attention levels, as described previously in our study [24]. EEG signals and CPM are potential biomarkers for understanding the neurophysiological mechanisms underlying pain modulation, particularly in the spinal cord injury (SCI) population where CPM is frequently impaired.

The strengths of this study are the sample size, the standardized recruitment and data collection protocols, and the use of well-known validated tools. The limitations are related to the cross-sectional design, which restricts the possibility of establishing causality between variables. Also, the absence of a control group limits the capacity to compare our findings with those of healthy subjects, reducing the generalizability of the results. However, our findings could identify associations and contribute to hypothesis generation for future studies. To address these limitations, longitudinal studies with a control group are necessary to provide more robust evidence regarding the temporal relationships between variables and to establish causality over time.

## 5. Conclusions

Our findings provide valuable insights into the neurophysiological mechanisms underlying SCI, contributing to the identification of associations and hypotheses for future research despite the limitations of a cross-sectional design and the lack of a control group. By integrating EEG biomarkers, we identified potential compensatory mechanisms that could inform personalized rehabilitation strategies. These insights lay the groundwork for longitudinal studies to explore temporal relationships and improve clinical outcomes in patients with SCI.

## Figures and Tables

**Table 1:** Demographic and clinical characteristics. *n* = 102.

Variables	Mean or *n* (sd or %)
Age (years)	41 (16)
Biological sex (%)	
Male	80 (87.9%)
Female	11 (12.1%)
Race	
White	41 (45.1%)
Black	10 (11%)
Mixed race	39 (42.9%)
Indigenous	1 (1.0%)
BMI (kg/m^2^)	24.52 (5.01)
Years of education	10.83 (4.49)
Handedness	
Right	85 (93.4%)
Left	6 (6.6%)
Smoking	
No	81 (89%)
Yes	10 (11%)
Alcohol consumption	
No	60 (65.9%)
Casually	25 (27.5%)
Yes	6 (6.6%)
Time of lesion (months)	19.1 (23.1)
Type of incapacity	
Tetraplegia	45 (44.12%)
Paraplegia	57 (55.88%)
Level of lesion	
Cervical	48 (47.06%)
Thoracic	41 (40.21%)
Lumbar	12 (11.76)
Sacral	1 (1.0%)
Walking index for spinal cord injury	4.98 (7.25)
Hospital anxiety scale	4.95 (3.97)
Hospital depression scale	3.98 (3.19)
MOCA	22.84 (4.45)
AIS grade, *n* = 88	
A	36 (40.9%)
B	14 (15.91%)
C	12 (13.64%
D	26 (29.55%)
E	none
CPM, kPa, *n* = 80	
Left tenar region	2.18 (0.04)
Right tenar region	2.07 (0.14)
TMS, *n* = 64	
MT of both hemispheres—mean	56.07 (12.04)
MEP 130% of MT both hemispheres—mean	0.97 (0.50)
ICI of both hemispheres—mean	2.59 (1.97)
ICF of both hemispheres—mean	1.91 (0.67)

BMI: body mass index, MOCA: Montreal cognitive assessment, AIS: ASIA impairment scale, kPa: kilopascal MT: motor threshold, MEP: motor-evoked potential, ICI: intracortical facilitation, ICF: intracortical inhibition. Continuous variables are presented with their mean (SD). Categorical variables are presented with their *n* (percentage).

**Table 2:** Resting-state EEG relative power (%), *n* = 86.

Band	Frontal (Mean + SD)	Central (Mean + SD)	Parietal (Mean + SD)
Delta	16.3 (6.2)	16.2 (6.0)	14.1 (5.1)
Theta	18.6 (7.7)	19.1 (7.4)	16.8 (7.5)
Alpha	46.7 (13.3)	43.2 (12.3)	50.9 (13.3)
Low alpha	25.9 (13.5)	22.3 (10.5)	27.3 (13.9)
High alpha	19.5 (12.4)	19.6 (11.0)	22.2 (13.8)
Beta	16.7 (7.2)	20.0 (8.1)	16.6 (7.1)
Low beta	10.4 (4.8)	12.5 (5.3)	10.4 (4.8)
High beta	6.4 (3.2)	7.3 (3.7)	6.1 (2.8)

**Table 3:** Resting state EEG—Hemisphere relative power (%), *n* = 86.

Band	Frontal (Mean + SD)		Central (Mean + SD)		Parietal (Mean + SD)	
	Left	Right	Left	Right	Left	Right
Delta	16.7 (6.7)	16.6 (6.4)	15.9 (6.0)	15.7 (6.1)	13.7 (6.2)	13.8 (6.2)
Theta	18.1 (7.4)	18.2 (7.4)	18.9 (7.4)	18.5 (7.5)	16.6 (7.6)	16.4 (7.6)
Alpha	46.9 (12.3)	46.5 (13.5)	43.0 (12.3)	44.2 (13.2)	51.0 (13.6)	51.8 (14.1)
Low alpha	25.8 (13.7)	25.2 (13.4)	21.8 (10.2)	22.8 (11.3)	27.2 (13.9)	28.0 (14.7)
High alpha	19.8 (12.8)	20.0 (12.6)	19.9 (11.0)	20.2 (11.7)	22.4 (14.0)	22.3 (14.2)
Beta	16.7 (7.4)	17.2 (0.7)	20.7 (8.6)	20.0 (8.6)	17.0 (7.5)	16.5 (7.4)
Low beta	10.2 (4.7)	10.5 (4.6)	12.9 (5.5)	12.7 (5.7)	10.7 (5.2)	10.4 (5.2)
High beta	6.4 (3.4)	6.6 (3.2)	7.6 (4.1)	7.2 (3.7)	6.2 (3.0)	6.1 (2.9)

**Table 4:** Baseline delta-band multivariate models according to ROI.

Variables	Beta Coefficient	*p*-Value	95% CI	R-Square
**Frontal region**				0.250
Depression	−0.01	0.036	−0.012 to 0.000	
Age	0.001	0.035	0.000 to 0.002	
Level of lesion	0.016	0.168	−0.007 to 0.040	
MOCA	−0.002	0.853	−0.027 to 0.022	
Years of education	0.007	0.503	−0.014 to 0.028	
**Parietal region**				0.205
ICI mean	0.010	0.022	0.001 to 0.019	
Age	0.001	0.086	0.000 to 0.002	
Biological sex	0.022	0.383	−0.028 to 0.071	
Level of lesion	0.016	0.366	−0.019 to 0.052	
Lesion time	−0.001	0.139	−0.002 to 0.000	

**Table 5:** Baseline delta-band multivariate models according to ROI.

Variables	Beta Coefficient	*p*-Value	95% CI	R-Square
**Frontal region**				0.322
MEP	−0.062	0.034	−0.118 to −0.005	
Biological sex	−0.024	0.456	−0.090 to 0.041	
Age	−0.105	0.008	−0.182 to −0.029	
Level of lesion	0.020	0.267	−0.017 to 0.056	
AIS	−0.036	0.132	−0.083 to 0.011	
**Central region**				0.293
MEP	−0.057	0.049	−0.114 to 0.000	
Biological sex	−0.025	0.452	−0.090 to 0.041	
Age	−0.105	0.008	−0.182 to −0.029	
Level of lesion	0.020	0.267	−0.017 to 0.056	
AIS	−0.047	0.054	−0.094 to 0.001	
**Parietal region**				0.405
MEP	−0.063	0.021	−0.116 to −0.010	
Biological sex	−0.016	0.607	−0.078 to 0.046	
Age	−0.062	0.077	−0.131 to 0.007	
Level of lesion	0.018	0.313	−0.018 to 0.054	
AIS	−0.038	0.093	−0.081 to 0.006	

**Table 6:** Baseline alpha-band multivariate models according to ROI.

Variables	Beta Coefficient	*p*-Value	95% CI	R-Square
**Frontal region**				0.210
MEP	0.111	0.008	0.030 to 0.192	
Biological sex	−0.008	0.869	−0.102 to 0.087	
Age	−0.082	0.167	−0.195 to 0.032	
Level of lesion	−0.038	0.184	−0.093 to 0.018	
**Central region**				0.136
MEP	0.087	0.040	0.004 to 0.170	
Biological sex	−0.005	0.915	−0.101 to 0.091	
Age	−0.056	0.342	−0.173 to 0.061	
Level of lesion	−0.018	0.530	−0.074 to 0.039	
**Parietal region**				0.231
MEP	0.110	0.010	0.027 to 0.193	
Biological sex	−0.023	0.633	−0.120 to 0.073	
Age	−0.106	0.075	−0.223 to 0.012	
Level of lesion	−0.016	0.549	−0.068 to 0.037	
SEPW total	−0.008	0.017	−0.015 to −0.001	0.157
Biological sex	−0.061	0.161	−0.147 to 0.024	
Age	−0.136	0.012	−0.242 to −0.030	

**Table 7:** Baseline high alpha-band multivariate models according to ROI.

Variables	Beta Coefficient	*p*-Value	95% CI	R-Square
**Frontal region**				0.134
CPM right hand	−0.012	0.049	−0.024 to −0.000	
Biological sex	−0.056	0.208	−0.145 to 0.032	
Age	−0.108	0.074	−0.227 to 0.011	
Level of lesion	0.023	0.2	−0.020 to 0.066	
**Parietal region**				0.181
CPM right hand	−0.016	0.018	−0.028 to −0.004	
Biological sex	0.067	0.161	−0.027 to 0.161	
Age	−0.148	0.033	−0.283 to −0.034	
Level of lesion	0.025	0.284	−0.020 to 0.070	

**Table 8:** Baseline frontal-asymmetry multivariate models.

Variables	Beta Coefficient	*p*-Value	95% CI	R-Square
**Delta band**				0.134
Depression	0.010	0.036	0.001 to 0.020	
Age	0.056	0.287	−0.048 to 0.160	
Lesion time	−0.002	0.040	−0.004 to 0.000	
**Alpha band**				0.132
Anxiety	−0.006	0.034	−0.011 to 0.000	
Age	−0.025	0.497	−0.097 to 0.047	
Lesion time	0.018	0.227	−0.011 to 0.046	
Level of lesion	0.017	0.267	−0.013 to 0.047	

**Table 9:** Summary of multivariate models by EEG bands and ROI.

Relative Power	Frontal	Central	Parietal
↑ Delta	↓ Depression		↑ ICI
	↑ Age		
↑ Delta asymmetry	↑ Depression		
	↓ Lesion time		
↑ Theta	↓ MEP	↓ MEP	↓ MEP
	↑ Age	↑ Age	↑ Age
↑ Alpha	↑ MEP	↑ MEP	↑ MEP
			↓ Sleepiness
			↓ Age
↑Alpha asymmetry	↓ Anxiety		
↑ High Alpha	↓ CPM right hand		↓ CPM right hand
	↓ Age		↓ Age

Only variables with statistical significance (*p* < 0.05) were included in this table.

## Data Availability

The data that support the findings of this study are available from the corresponding author upon reasonable request due to privacy reasons.

## Appendix A

EEG variables were treated as dependent variables, and other neurophysiological, clinical, and demographic data were treated as independent variables. First, to ensure the integrity of our regression analysis, we followed the four assumptions of linearity, homoscedasticity, independence, and normally defended by Osborne and Waters (2002) [51]: (i) normality: we used Histogram and Shapiro–Wilk tests to assess data distribution; (ii) independence: we used the Durbin–Watson statistic to ensure the absence of autocorrelation in the residuals of the regression models; (iii) linearity: we assessed the assumption by visually comparing the scatterplot of each independent variable and a superimposed regression line; (iv) homoscedasticity: plots of residuals against predicted values were inspected; (v) independence: this helped us ensure that there was no autocorrelation in the residuals of the regression models. Also, values greater than 3 SDs away from the mean scores of the dependent or independent variables were labeled as outliers.

Second, we conducted a linear univariate analysis to identify the independent variables associated with EEG-dependent variables. The independent variables considered in our analysis are related to (i) demographic variables: age, biological sex, race, years of study, and handedness; (ii) clinical variables: BMI, level of injury, time of lesion, depression and anxiety scales, AIS, and MOCA; and (iii) other neurophysiological variables: CPM and TMS parameters. In the univariate analysis, we selected the variables with a *p*-value < 0.20 to be included in the multivariate analysis. Statistical significance was set at *p* < 0.05.

Finally, we employed a multicriteria approach to search for confounders to select our final multivariate modes: (i) based on the literature supporting physiological plausibility, (ii) considering changes in β coefficients greater than 10% when a variable was added or removed, and (iii) selecting the variables that would provide the best fit based on the previous the four assumptions presented by Osborne and Waters (2002) [51]. In addition, we examined the relationship between the primary predictor variables and demographic and clinical factors incorporated into the final models. Besides, to determine frontal asymmetry, the delta and alpha relative powers of the frontal left and right hemispheres were natural log-transformed, and the asymmetry score was estimated by subtracting the value of the left from the right hemisphere (for instance, frontal delta asymmetry score = natural log-transformed relative power of frontal delta right—natural log-transformed relative power of frontal delta left), as suggested by Tomarken et al. [52].

We used STATA^®^ 17.0 for all statistical analyses. Hence, considering the nature of this exploratory analysis, our regression analysis was limited to testing for associations between the dependent and independent variables and did not predict the impact of the independent variables on EEG power values. Therefore, our findings can be interpreted as statistical tests of correlation where the EEG variables were initially tested for associations with clinical, demographic, and other neurophysiological variables.
